# How Many Sexes? How Many Genders?

**DOI:** 10.1007/s10508-025-03209-z

**Published:** 2025-09-10

**Authors:** Alex Byrne

**Affiliations:** Department of Linguistics and Philosophy, Massachusetts Institute of Technology, Cambridge, MA 02139 USA

## How Many Sexes?

Two (Parker, 2011).

## How Many Genders?

The British philosopher and public intellectual C. E. M. Joad was a regular panelist on the BBC radio show *The Brains Trust* during and after the Second World War. He often began an answer to listeners’ questions with his catchphrase “It all depends what you mean by…,” which caught on throughout the country (Ayto & Crofton, 2011). If any question deserves Joad’s catchphrase, it is “How many genders?”

One meaning of “gender” is “sex,” a usage which dates to the fifteenth century (Oxford University Press, 2025). If that is the intended interpretation, then the answer to the question is “Two” (see above). Another meaning was introduced in Robert Stoller’s 1968 book *Sex and Gender*:

Gender is the amount of masculinity or femininity found in a person, and, obviously, while there are mixtures of both in many humans, the normal male has a preponderance of masculinity and the normal female a preponderance of femininity. (Stoller 1968, pp. 9–10)

If we take this use of “gender” seriously, “How many genders?” is not a clear question. Stoller himself answers “Two,” writing of “two biologic sexes, male and female, with two resultant genders, masculine and feminine” (p. 29). However, in the spirit of Stoller, one could equally well take each specific “mixture” of masculinity and femininity to be a “gender,” in which case the answer to our question is “Indefinitely many.”

In fact, every other use of “gender” makes counting genders problematic. Take, for example, the World Health Organization:

Gender refers to the characteristics of women, men, girls and boys that are socially constructed. This includes norms, behaviours and roles associated with being a woman, man, girl or boy, as well as relationships with each other. As a social construct, gender varies from society to society and can change over time. (World Health Organization, n.d.)

The WHO does not explain what it is for a characteristic to be “socially constructed.” But presumably “primary caregiver” and “dress-wearer” are examples of socially constructed characteristics (norms, behaviors, roles), as the WHO understands them. Taking the main responsibility for the care of children is associated with being a woman in every human society; wearing a dress is associated with being a woman only in some societies. If each such characteristic is a “gender,” then there are an awful lot of them.

The American Psychological Association offers a similar account, albeit one with even more problems. According to the APA’s Dictionary of Psychology, “gender” refers to:

the socially constructed roles, behaviors, activities, and attributes that a given society considers appropriate for different genders. (American Psychological Association, 2023)

Like the WHO, the APA does not explain what “socially constructed” means. Worse, the APA’s definition of “gender” uses the word itself, making the definition circular. Another example of circularity is in the current *Diagnostic and Statistical Manual of Mental Disorders*:

*Gender* is used to denote the public, sociocultural (and usually legally recognized) lived role as boy or girl, man or woman, or other gender. (American Psychiatric Association, 2022, p. 511)

Sometimes “gender” is used as an abbreviation for “gender identity”—in its contemporary guise, a dubiously coherent notion (see Byrne, 2023a, 2023b; Haig, 2023; Janssen, 2023). Turban (2024) has recently estimated the number of gender identities to be “nearly infinite” (p. 50).

In short, “gender is a quagmire” (Quintana & Pfaus, 2024, p. 2964). The current fashion for “gender/sex” only serves to plunge us deeper into the ooze. According to DuBois and Shattuck-Heidorn (2021), “gender/sex” (or “sex/gender”) means “the biosocial entwinement of sex and gender” (Table 1, p. 3). “Gender” is explained as “culturally contextualized social and structural experiences as well as expressions of identity” (Table 1). Biosocially entwining these experiences and expressions with sex apparently yields more than one result, because Dubois and Shattuck-Heidorn write of “people of all genders/sexes” (p. 10). How many genders/sexes are there, and what is an example of one? DuBois and Shattuck-Heidorn do not say. A new paper by Fausto-Sterling (2025) mentions “the baseline assumption that gender/sex is dichotomous or binary” (p. 1), an assumption which Fausto-Sterling evidently wishes to question. Since Fausto-Sterling never explains what gender/sex is supposed to be, the baseline assumption is obscure.

“Gender/sex” does receive a detailed definition from van Anders (2015): “Whole people/identities and/or aspects of women, men, and people that relate to identity and/or cannot really be sourced specifically to sex or gender” (Table 2). This is hard to understand. Confusingly, “trans” and “transgender” are given as examples of “gender”; “trans” and “transsexual” as examples of “sex”; and “trans woman” and “trans man” as examples of “gender/sex” (Table 2, p. 1181).

As the citation in the first section indicates, “sex” is indispensable. The moral of the second section is that sexology and science in general would be better off without “gender.”
